# The Hookworm Blues: We Still Got ‘em

**DOI:** 10.4269/ajtmh.17-0683

**Published:** 2017-11-08

**Authors:** John W. Sanders, Karen A. Goraleski

**Affiliations:** 1 ASTMH Clinical Group President, Oakbrook Terrace, Illinois;; 2 ASTMH Executive Director, Oakbrook Terrace, Illinois

Hookworm in your body

And your food don't do you no good

Same way with a rounder come in a nice neighborhood

“Hookworm Blues,” Blind Arthur Blake

Published June, 1929

Two articles published in this issue of the American Journal of Tropical Medicine and Hygiene (AJTMH) address a similar theme. McKenna et al.^1^ report a study evaluating the prevalence of intestinal parasites in a cross-sectional cohort of 24 households in rural Alabama. The authors excluded subjects with any international travel, and found that of the 55 stool specimens tested by molecular methods, 34.5% were positive for hookworm (*Necator americanus*), and 7.3% were positive for *Strongyloides stercoralis*. Guerrero-Wooley et al.^2^ report the case of a man from rural Appalachia with no international travel who presented with *Strongyloides* hyperinfection during his treatment of chronic lymphocytic leukemia. The articles serve as sobering reminders that “tropical medicine and hygiene” has relevance even within the borders of the United States, and especially for those providing clinical care in the impoverished rural South, we must remember to consider the presence of endemic tropical diseases.

Many Americans are unaware that tropical diseases in the classical sense were historically endemic throughout North America after the introduction of various diseases by European settlers and their African slaves. Malaria was prevalent from the Gulf of Mexico to northern Canada by the seventeenth century.^3^ Dr. Benjamin Rush, a signer of the Declaration of Independence, wrote classic descriptions of both dengue and yellow fever epidemics in Philadelphia.^4,5^ These diseases traveled with settlers as they made their westward migration but never became as well established in other parts of the country.^3,6^ While there were short periods of re-establishment of malaria in the North after Federal soldiers returned home from the South after the Civil War, and a yellow fever outbreak moved from New Orleans as far north as Ohio in 1878, these had largely become southern diseases by the mid-nineteenth century.^3,6,7^ Malaria became established as a normal part of rural life in much of the deep South, where agricultural practices maintained persistent exposure to the vector.^3,6^ Outbreaks of dengue were fairly common in port cities throughout the South and began spreading across Texas in the latter half of the nineteenth century.^5^ Outbreaks of yellow fever, which occurred with frightening regularity, were especially problematic for port cities in the South, as the risk of yellow fever and subsequent quarantine of goods created a negative economic incentive for merchants to use those ports.^7^ Although transmission of some diseases was in decline, thanks to the “Great Sanitary Awakening” of the nineteenth century,^3^ the discovery of the role of mosquitoes in the transmission of yellow fever and malaria led to more purposeful and successful efforts that dramatically decreased transmission of these diseases. The last outbreak of yellow fever in a U.S. city was in New Orleans in 1905,^7^ the last recorded outbreaks of dengue were in Louisiana in 1945,^5^ and the United States was subsequently declared free from the endemic transmission of malaria in 1951.^3,6^

While these classic tropical diseases are prevalent, no infection is more associated with the American South than hookworm.^7,8^ *Necator americanus*, the New World hookworm, a common parasite in Africa during the time of the European settlers, was probably introduced to the South through the slave trade. Hookworm, *Strongyloides*, and other geohelminths found a hospitable environment, characterized by a warm, moist climate; sandy or loamy soils which provided burrowing hookworm larvae protection from the sun; and poor hygiene and waste disposal practices. Similarly, in the Appalachians, hookworm found refuge in mines and in the muddy, clay soil. Although certainly hyperprevalent for generations, hookworm was not recognized as a problem in the American South until 1902, when Charles Stiles recognized the similarity of symptoms between many hookworm patients from Europe and the southern United States.^7,8^ Subsequent epidemiologic studies supported by John D. Rockefeller discovered hookworm prevalence of about 40% throughout the rural South. It is now generally accepted that the anemia and lassitude associated with a heavy worm burden contributed significantly to decreased productivity in the region.^8,9^ Control efforts initiated by the Rockefeller Sanitary Commission for the Eradication of Hookworm Disease and subsequently taken up by local and state governments spanned 11 states, included treatment of more than 400,000 people, and expanded education to healthcare providers, teachers, and the general public about infection risks, leading to the promotion of prevention measures such as the construction of latrines and the routine wearing of shoes.^8,9^ Prevalence of infection dropped considerably over the next several decades, but studies in the 1950s and 1960s found that hookworm prevalence of up to 15% still existed in poor populations in south Alabama and eastern Kentucky.^7^ Despite this persistence, there is good evidence to suggest that decreased worm burden contributed to the educational improvements and economic growth seen throughout much of the South in the last century.^8,9^

We are now regularly and appropriately reminded that tropical infections pose a health risk to the United States and that additional infections can become re-established here.^10^ After being eliminated from the South, dengue re-emerged and autochthonous cases are now regularly reported from Texas and Florida.^5^ West Nile virus has become endemic in the United States; chikungunya virus and Zika virus have each been locally transmitted, and there are warnings about the potential for a yellow fever outbreak.^11^ Parasitic infections such as malaria, neurocysticercosis, Chagas disease, and geohelminth infections can be found commonly in immigrants and international travelers.^3,12–16^ There is a key difference in these concerns and that which is raised by the two articles in this issue.^1,2^ Those other examples represent diseases presumed to have been eliminated from within our borders, whereas these articles demonstrate that we never completely eliminated geohelminth infections from the South.

McKenna et al.^1^ have presented a very interesting study in which they evaluated households in Lowndes County, Alabama, for the presence of nine intestinal parasites using both standard and molecular techniques. We should not exaggerate the results of the study. The study population was small, involving only 24 households and 55 subjects. The positive results were largely established with highly sensitive molecular testing, with little or no confirmation by microscopy, likely reflecting a low parasite burden. Still, and remarkably, more than a third (19/55) of these patients tested positive for hookworm, and 4/55 (7.3%) were positive for *Strongyloides*. The explanation for these high numbers was self-evident from the associated questionnaires. Adequate septic systems were uncommon. Instead, a rudimentary system involving piping from the toilet emptying into an open area, such as a ditch, just a few meters distant from housing, appeared common. This is not even as protective against fecal contamination as a reasonably dug outhouse. This finding highlights a common observation about these infections: they are more specifically associated with poverty than with the tropics.^17^

Guerrero-Wooley et al.^2^ provide the details of a case of an unfortunate 64-year-old man who was born and raised in Appalachia and who succumbed to hyperinfection with *Strongyloides*.^2^ The clinical case itself is not unusual, as there are many reports of patients from endemic regions presenting with *Strongyloides* hyperinfection after becoming immunosuppressed. For example, Glenn et al also report in this issue a challenging case of recurrent *Strongyloides* hyperinfection in a military veteran with multiple myeloma who was born in Puerto Rico.^18^ While the prevalence of *Strongyloides* infection is likely much lower in Puerto Rico than other parts of Latin America, it is reasonably described as an endemic region.^19^ Was the patient presented by Guerrero-Wooley also from an endemic region? It is a common teaching point that *Strongyloides* infection is prevalent in Appalachia, but minimal recent epidemiologic data are available to back up this claim. In 2014, Russell et al.^18^ reported 1.9% *Strongyloides* seropositivity in an Appalachian region of Kentucky, but no clinical evidence of current infection was presented. Although the patient described in this issue was potentially infected many years ago, his case adds some clinical context to that earlier serosurvey and reinforces recommendations to further assess *Strongyloides* prevalence.

This editorial is meant to draw attention to a central issue raised in both of the new papers discussed above and to address how the American Society of Tropical Medicine and Hygiene (ASTMH) can help from a training and policy perspective. Many of the diseases that have historically been considered tropical diseases are more accurately diseases of poverty. The American South has made tremendous progress in dealing with both tropical diseases and poverty. We must therefore confess tremendous surprise to see such a high prevalence of infection for hookworm and *Strongyloides* in Lowndes County, Alabama. This is not a remote or isolated area of the country. Its county seat courthouse is merely 24 miles from the State Capitol Building. The patient from Appalachia is a neighbor to retirement homes that boast “million dollar views,” but he and many of his friends and family are living a different economic reality. For clinicians everywhere, but in particular for those in the South, we must maintain a heightened awareness that tropical diseases have been, and in the setting of abject poverty, likely still are endemic here. We need to keep the possibility of these infections in mind as we provide care for our patients, to assure that our students are receiving appropriate training in parasite infections and other diseases of poverty, and to strive to improve our understanding of the persisting problem of tropical infections in the United States.

The ASTMH—through the AJTMH, the Annual Meeting, a Clinical Update Course, and a Certificate of Knowledge in Clinical Tropical Medicine and Travelers’ Health (CTropMed^®^)—offers avenues for this specialized training. In spite of a globally mobile population and significant rates of poverty, courses on tropical diseases and diseases of poverty have been slowly trimmed back in today’s medical school curriculums, thus positioning ASTMH’s role in this arena as more needed than ever. On the policy front, the ASTMH advocates for evidence-based policies in the United States that reference the role of poverty in persisting tropical diseases. These studies reaffirm what has driven this society since 1903—a world free of tropical infectious diseases that have disproportionately affected people living in poverty.
